# Comparative Effect of Statins and Omega-3 Supplementation on Cardiovascular Events: Meta-Analysis and Network Meta-Analysis of 63 Randomized Controlled Trials Including 264,516 Participants

**DOI:** 10.3390/nu12082218

**Published:** 2020-07-25

**Authors:** Tung Hoang, Jeongseon Kim

**Affiliations:** Department of Cancer Biomedical Science, National Cancer Center Graduate School of Cancer Science and Policy, Goyang 10408, Korea; 75256@ncc.re.kr

**Keywords:** Cardiovascular event, statin, omega-3, network meta-analysis

## Abstract

Statins and omega-3 supplementation have been recommended for cardiovascular disease prevention, but comparative effects have not been investigated. This study aimed to summarize current evidence of the effect of statins and omega-3 supplementation on cardiovascular events. A meta-analysis and a network meta-analysis of 63 randomized controlled trials were used to calculate pooled relative risks (RRs) and 95% confidence intervals (CIs) for the effects of specific statins and omega-3 supplementation compared with controls. Overall, the statin group showed significant risk reductions in total cardiovascular disease, coronary heart disease, myocardial infarction, and stroke; however, omega-3 supplementation significantly decreased the risks of coronary heart disease and myocardial infarction only, in the comparison with the control group. In comparison with omega-3 supplementation, pravastatin significantly reduced the risks of total cardiovascular disease (RR = 0.81, 95% CI = 0.72–0.91), coronary heart disease (RR = 0.75, 95% CI = 0.60–0.94), and myocardial infarction (RR = 0.71, 95% CI = 0.55–0.94). Risks of total cardiovascular disease, coronary heart disease, myocardial infarction, and stroke in the atorvastatin group were statistically lower than those in the omega-3 group, with RRs (95% CIs) of 0.80 (0.73–0.88), 0.64 (0.50–0.82), 0.75 (0.60–0.93), and 0.81 (0.66–0.99), respectively. The findings of this study suggest that pravastatin and atorvastatin may be more beneficial than omega-3 supplementation in reducing the risk of total cardiovascular disease, coronary heart disease, and myocardial infarction.

## 1. Introduction

Cardiovascular diseases (CVDs) are characterized by any disorders relating to the heart or blood vessels [1], including coronary heart disease (CHD), cerebrovascular disease, stroke, peripheral vascular disease, rheumatic and congenital heart diseases, and venous thromboembolism [2]. It has been reported that the burden of CVDs has remained high over the past decades, with 75% of overall CVD deaths occurring in low- to middle-income countries [3]. In general, the prevalence of CVDs is closely related to various risk factors, such as hypertension, overweight and obesity, dyslipidemia, and diabetes [4,5]. According to recent guidelines of the American College of Cardiology/American Heart Association (ACC/AHA) regarding the primary prevention of CVD, individuals aged 40 to 75 years with diabetes mellitus and low-density lipoprotein-cholesterol (LDL-C) ≥70 mg/dL or those with hypercholesterolemia (LDL-C ≥190 mg/dL) regardless of CVD risk are recommended with moderate to high-intensity statins [6,7]. Additionally, according to the European Society of Cardiology and the European Atherosclerosis Society (ESC/EAS) guidelines, high-risk individuals with LDL-C ≥190 mg/dL or triglyceride (TG) >200 mg/dL are also suggested with statin therapy [8,9].

Although findings from a meta-analysis of 19 randomized controlled trials (RCTs) showed the protective effect of statins on CVD events among a high-risk population compared with a placebo or non-statin interventions [10], updated evidence from recent RCTs is required. In addition, a recent meta-analysis of 13 RCTs found marine omega-3 supplementation to be associated with a decreased risk of CVD events in a dose-response manner [11], though the analysis was restricted to only certain RCTs [12,13,14,15,16]. Overall, the pairwise effects of statins and omega-3 supplementation on CVD prevention have not been investigated. Therefore, this study aims to systematically summarize current evidence of the effect of statins and omega-3 supplementation through a meta-analysis and to elucidate the comparative efficacy of statins and omega-3 supplementation in the prevention of cardiovascular events through a network meta-analysis (NMA) of RCTs.

## 2. Methods

### 2.1. Literature Search

We searched RCTs from previously published systematic reviews or meta-analyses of statin use or omega-3 supplementation in PubMed from its inception to January 2020. The search keywords were statin (statins or *statin), omega-3 (fish oil) supplementation (supplement or supplements), cardiovascular disease (cardiovascular diseases), systematic review, and meta-analysis.

### 2.2. Eligibility Criteria

Two review authors (T.H. and J.K.) independently performed the search and assessed the eligibility of studies. We included RCTs reporting the comparative efficacy of a statin versus a statin, a statin versus an omega-3 supplementation, a statin versus a control, or an omega-3 supplementation versus a control for the prevention of total CVD, CHD, myocardial infarction (MI), and stroke. We considered seven currently available HMG-CoA reductase inhibitors: atorvastatin, fluvastatin, lovastatin, pitavastatin, pravastatin, rosuvastatin, and simvastatin.

The following exclusion criteria were used: outcome data or full text not available; follow-up duration less than 1 year; source of omega-3 from dietary intake; or placebo containing omega-6. Studies investigating the effect of a high dose compared with a low dose of the same treatment or comparing the intervention with an active drug were also excluded. For each RCT, the following information was extracted: study name, publication year, mean or median age (years) and follow-up time (years); the percentages of subjects with a history of MI, hypertension, diabetes, and smoking; treatment name, sample size, number of each cardiovascular event, and baseline and achieved serum concentrations of total cholesterol (TC), TG, high-density lipoprotein cholesterol (HDL-C), and LDL-C for each treatment arm.

### 2.3. Statistical Analysis

We first investigated the association between statins and omega-3 supplementation and the risk of total CVD, CHD, MI, and stroke in a meta-analysis of direct evidence. Subgroup analyses for statins and sensitivity analyses using both fixed-effect and random-effects models were also performed. The Higgins I^2^ metric [17] was used to measure heterogeneity across individual studies. Additionally, we performed meta-regression analysis to determine whether the effects of total statins and omega-3 supplementation on cardiovascular event prevention were modulated due to their lipid-lowering effect. In particular, the lipid lowering effect was identified as the absolute difference (mg/dL) in lipid changes (= achieved level—baseline level) between the intervention arm and the comparison arm. Weighted mean difference (WMD) and 95% CI were calculated for the pooled effect of statins and omega-3 supplementation on LDL-C reduction. We further applied a generalized additive model using the ‘mgcv’ package [18] to access the non-linear association between cardiovascular event prevention effect and LDL-C reduction in statins and omega-3 supplementation arm separately.

Relative risks (RRs) and 95% confidence intervals (CIs) for the comparative efficacy of statins, omega-3 supplementation, and control were computed in the NMA. As the prior probability was not established in the study hypothesis, we performed the NMA with the frequentist approach to combine direct and indirect evidence [19]. A funnel plot was utilized to assess publication bias [20], and a contribution plot was applied to examine the influence of direct estimates on network summary effects [21]. Stata SE version 14.0 (StataCorp, College Station, TX, USA) and R software (R Foundation for Statistical Computing, Vienna, Austria) (version 3.5.2) were used for all statistical analyses.

## 3. Results

### 3.1. Study Characteristics

**Figure 1:**
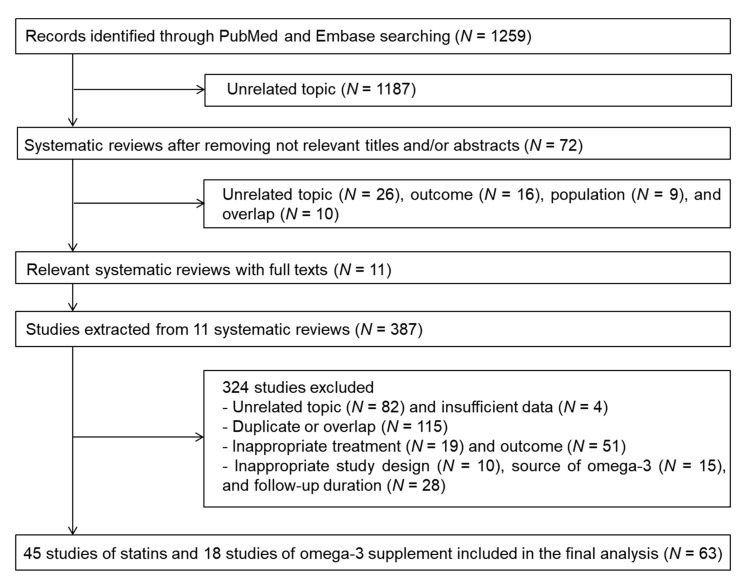
Flow diagram of the systematic review.

In total, 1259 potentially relevant records were assessed (Figure 1); 11 meta-analyses [11,22,23,24,25,26,27,28,29,30,31] were reviewed, and eligible RCTs were extracted. Of the 387 extracted RCTs, 324 were excluded for the following reasons: unrelated topic (*N* = 82), insufficient data (*N* = 4), duplicate publication or population overlap (*N* = 115), inappropriate treatment (*N* = 19), non-target outcome (*N* = 51), other study design (*N* = 10), external source of omega-3 (*N* = 15), and duration of follow-up (*N* = 28). Ultimately, 45 RCTs of statins and 18 RCTs of omega-3 supplementation involving 264,516 adults were included in the meta-analysis and NMA [12,13,14,15,16,32,33,34,35,36,37,38,39,40,41,42,43,44,45,46,47,48,49,50,51,52,53,54,55,56,57,58,59,60,61,62,63,64,65,66,67,68,69,70,71,72,73,74,75,76,77,78,79,80,81,82,83,84,85,86,87,88].

Table 1 summarizes the characteristics of the included RCTs. The median age of the participants was 62.6 years, and the follow-up duration was 3.7 years; the rates of a history of MI, hypertension, diabetes, and smoking were 24.8%, 50.6%, 18.9%, and 38.6%, respectively. Additionally, the lipid profiles of TC, TG, HDL-C, and LDL-C of study participants from individual RCTs at baseline and differences in the change of serum lipid concentrations during the follow-up are presented in Table 2.

### 3.2. Meta-Analysis of Direct Estimates

In comparison with the control group, the pooled direct estimates of statins or omega-3 supplementation and the risk of CVD events are summarized in Table 3. Overall, the levels of significant findings were equivalent in both the fixed-effects and random-effect models, except for the association between atorvastatin or rosuvastatin and the risk of stroke. The total statin and omega-3 groups exhibited reduced risks of total CVD (RR = 0.89, 95% CI = 0.85–0.94), CHD (RR = 0.81, 95% CI = 0.75–0.89), MI (RR = 0.78, 95% CI = 0.78–0.85), and stroke (RR = 0.91, 95% CI = 0.85–0.98).

In subgroup analysis by statins, total statins were associated with decreased risks of total CVD, CHD, MI, and stroke, with RRs (95% CIs) of 0.81 (0.76–0.86), 0.70 (0.62–0.77), 0.69 (0.61–0.78), and 0.85 (0.79–0.82), respectively. Individually, atorvastatin and pravastatin had significant effects on all outcomes. Furthermore, rosuvastatin was associated with significantly reduced risks of CHD and MI, and lovastatin was associated with a significant risk reduction in total CVD. Fewer stroke events occurred in the simvastatin group than in the control group, and fluvastatin was observed to reduce total CVD. Moreover, omega-3 supplementation was associated with a 19% reduced risk of CHD (RR = 0.81, 95% CI = 0.75–0.89) and an 11% reduced risk of MI (RR = 0.89, 95% CI = 0.80–0.99).

**Figure A1:**
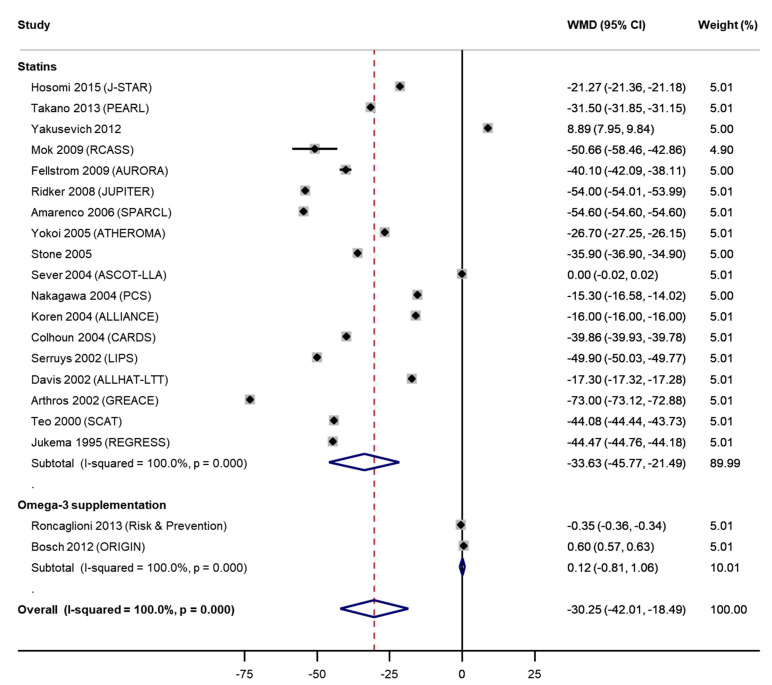
Meta-analysis for LDL-C lowering effect of statins and omega-3 supplementation. WMD, weighted mean difference; CI, confidence interval; LDL-C, low-density lipoprotein cholesterol

**Figure A2:**
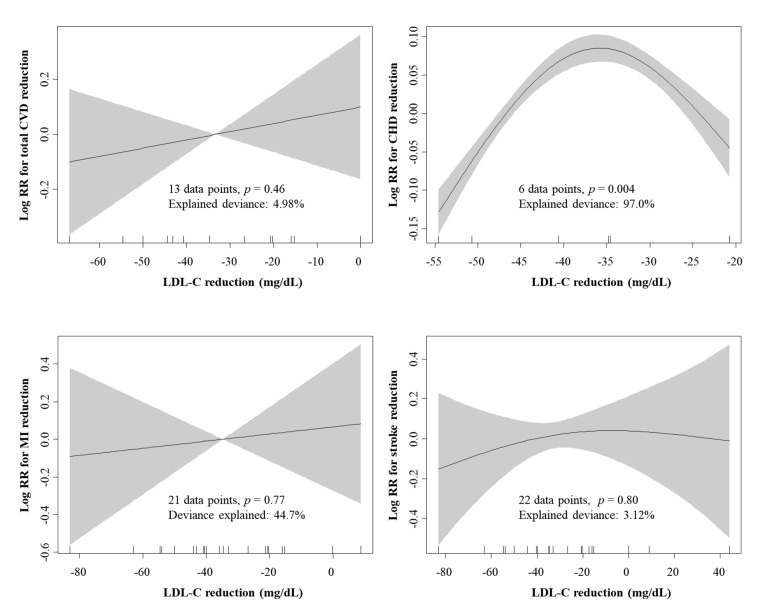
Non-linear association between cardiovascular event reduction and LDL-C lowering effects of statins. Cardiovascular events include total cardiovascular disease (CVD), coronary heart disease (CHD), myocardial infarction (MI), and stroke. RR, relative risk; CVD, cardiovascular disease; CHD, coronary heart disease; MI, myocardial infarction; LDL-C, low-density lipoprotein cholesterol.

**Figure A3:**
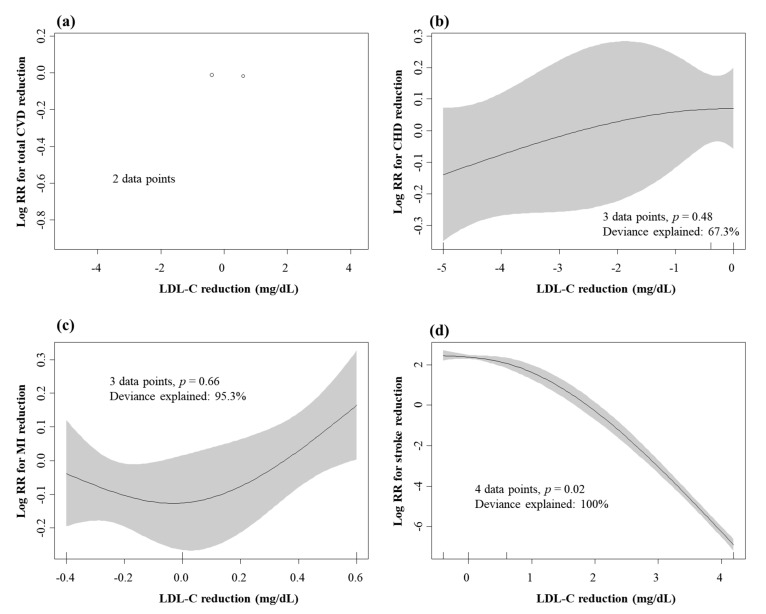
Non-linear association between cardiovascular event reduction and LDL-C lowering effects of omega-3 supplementation. Cardiovascular events include (**a**) total cardiovascular disease (CVD), (**b**) coronary heart disease (CHD), (**c**) myocardial infarction (MI), and (**d**) stroke. RR, relative risk; LDL-C, low-density lipoprotein cholesterol.

The meta-regression coefficients for the association between the lipid-lowering effect and risk reduction of cardiovascular events are presented in Table 4. The size of TC reduction achieved with overall statins and omega-3 supplementation was significantly associated with the size of total CVD and CHD reduction effect, with coefficients (*p*-values) of 0.0046 (<0.001) and 0.0042 (0.047), respectively. Additionally, total CVD and CHD reduction effect might be driven by per unit change in LDL-C level, with decreases of 0.0034 (*p* = 0.004) and 0.0044 (*p* = 0.047) in log (RRs) for total CVD and CHD per 1 mg/dL LDL-C lowering effect of statins and omega-3 supplementation, respectively. The combined meta-analysis of 20 effect sizes showed a significant reduction in LDL-C concentration following administration of statins (WMD, −33.63 mg/dL; 95% CI, −45.77 to −21.49 mg/dL; Figure A1), but not omega-3 supplementation (WMD, 0.12; 95% CI, −0.81 to 1.06 mg/dL; Figure A1). Relationships between cardiovascular event reduction and LDL-C lowering effects by subgroup for statins and omega-3 supplementation are presented in Figure A2 and Figure A3. CHD reduction effect of statins (*p* = 0.004) and stroke reduction effect of omega-3 supplementation (*p* = 0.02) were observed to be non-linearly associated with the LDL-C lowering effect, with the explained deviance of over 97%.

The coefficient from the meta-regression model represents the change in the log-transformed relative risk for every increase of one mg/dL in serum lipid concentrations.

### 3.3. Network Meta-Analysis of Direct and Indirect Estimates

**Figure 2:**
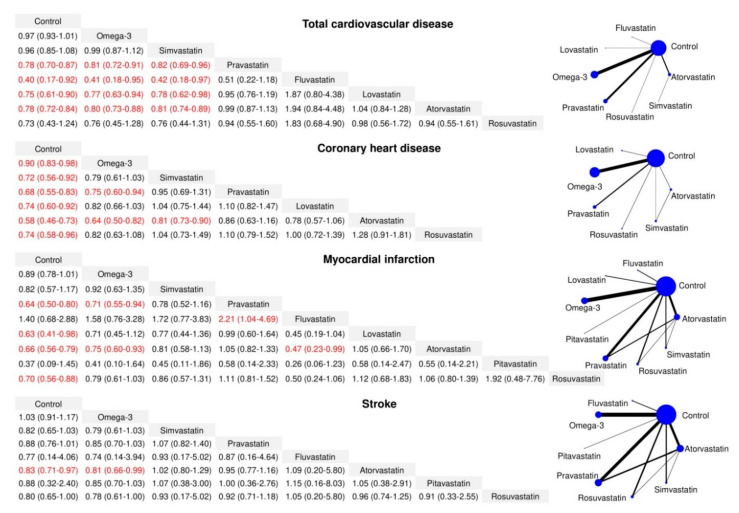
Network meta-analysis of statin, omega-3, and control in the risk of different cardiovascular events.

Figure 2 presents the comparative effects among statins, omega-3 supplementation, and controls with regard to the risk of CVD events. Compared with findings from the meta-analysis of direct evidence, a significant effect of simvastatin (RR = 0.72, 95% CI = 0.56–0.92) on CHD was also observed, whereas the network estimates did not reveal any significant effects of pravastatin (RR = 0.88, 95% CI = 0.76–1.01), simvastatin (RR = 0.82, 95% CI = 0.65–1.03), or omega-3 supplementation (RR = 1.03, 95% CI = 0.91–1.17) on the risk of stroke.

Pravastatin had a significantly lower risk of total CVD (RR = 0.81, 95% CI = 0.72–0.91), CHD (RR = 0.75, 95% CI = 0.60–0.94), and MI (RR = 0.71, 95% CI = 0.55–0.94) than omega-3 supplementation. The risks of total CVD, CHD, MI, and stroke in the atorvastatin group were lower than those in the omega-3 group, with RRs (95% CIs) of 0.80 (0.73–0.88), 0.64 (0.50–0.82), 0.75 (0.60–0.93), and 0.81 (0.66–0.99), respectively. Additionally, the risk of total CVD in the fluvastatin and lovastatin groups was lower than that in the omega-3 group, with RRs (95% CI) of 0.41 (0.18–0.95) and 0.77 (0.63–0.94), respectively.

Among statins, pravastatin, fluvastatin, lovastatin, and atorvastatin were found to be associated with lower risks of total CVD than simvastatin, with RRs (95% CIs) of 0.82 (0.69–0.96), 0.42 (0.18–0.97), 0.78 (0.62–0.98), and 0.81 (0.74–0.89), respectively. The risk of CHD in the atorvastatin group was also 19% lower than that in the simvastatin group (RR = 0.81, 95% CI = 0.73–0.90). Fluvastatin was associated with a higher MI risk than pravastatin, with RR = 2.21 and 95% CI = 1.04–4.69.

Data are presented as the relative risk and 95% confidence interval of row-defined intervention versus column-defined comparison for the risk of different cardiovascular events and with corresponding network plots of available direct comparison. The size of nodes is proportional to the number of studies including the respective treatments. The thickness of the edges is proportional to the mean control group risk for comparisons between control and active treatment groups.

### 3.4. Publication Bias and Contribution Plot

**Figure 3:**
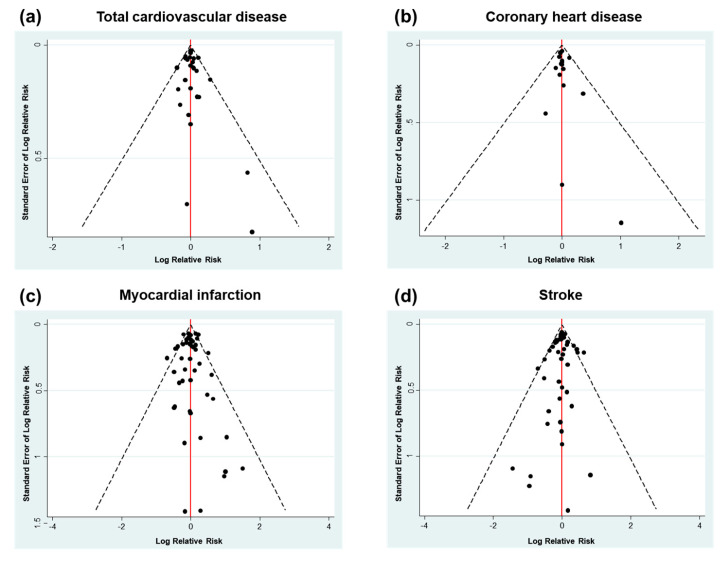
Funnel plot for publication bias according to (**a**) total cardiovascular disease, (**b**) coronary heart disease, (**c**) myocardial infarction, and (**d**) stroke.

**Figure A4:**
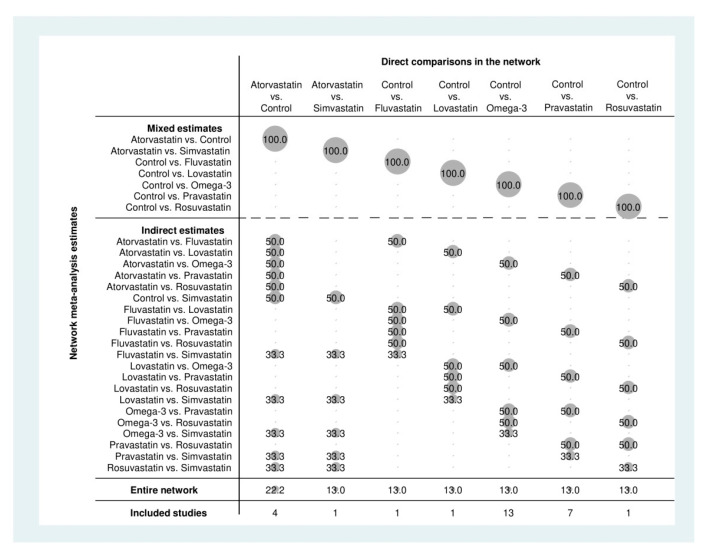
Contribution plot for the total cardiovascular disease network. The size of the square is weighted as the percentage (as number) of the contribution of the direct estimate (horizontal axis) to the network estimate (vertical axis).

**Figure A5:**
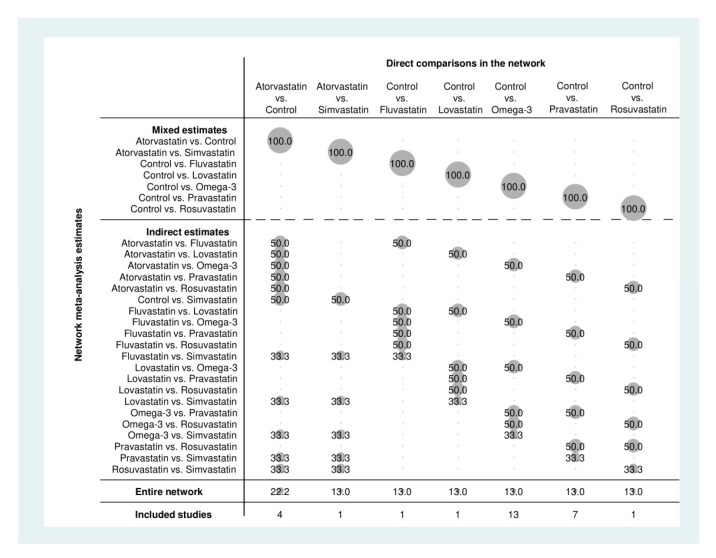
Contribution plot for the coronary heart disease network. The size of the square is weighted as the percentage (as number) of the contribution of the direct estimate (horizontal axis) to the network estimate (vertical axis).

**Figure A6:**
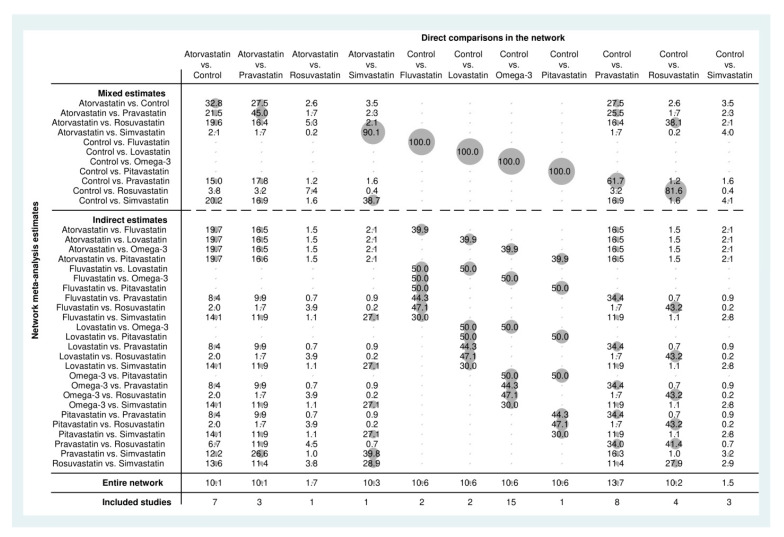
Contribution plot for the myocardial infarction network. The size of the square is weighted as the percentage (as number) of the contribution of the direct estimate (horizontal axis) to the network estimate (vertical axis).

**Figure A7:**
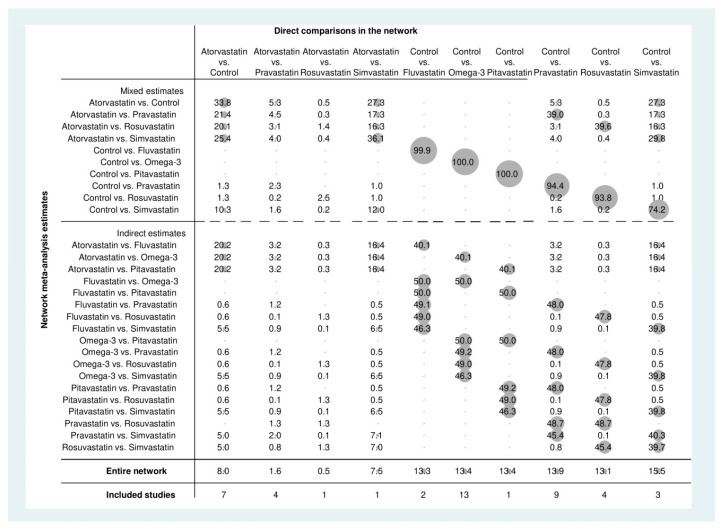
Contribution plot for the stroke network. The size of the square is weighted as the percentage (as number) of the contribution of the direct estimate (horizontal axis) to the network estimate (vertical axis).

Funnel plot asymmetry indicating potential publication bias was not observed for different outcomes of CVD events (Figure 3). The contribution to mixed estimates and the influence on indirect estimates of direct estimates are presented in Figure A4, Figure A5, Figure A6 and Figure A7. Overall, most of the direct comparisons contributed nearly 100% to the same mixed comparisons in terms of total CVD (Figure A4) and CHD outcomes (Figure A5), except for control versus simvastatin. In contrast, approximately half of the direct comparisons did not contribute to the same mixed comparisons in terms of MI (Figure A6) and stroke outcomes (Figure A7).

### 3.5. Treatment Ranking

Fluvastatin had the highest probability of being the primary intervention for total CVD (85.4%) and stroke (42.2%), whereas atorvastatin and pitavastatin had the highest probabilities in terms of CHD (78.1%) and MI (72.7), respectively (Table 5). The surface under the cumulative ranking curve (SUCRA) values of cumulative ranking probability, which accounts for the uncertainty of spuriously high ranks, suggested better ranks for fluvastatin, atorvastatin, pitavastatin, and rosuvastatin in the prevention of total CVD (0.9), CHD (1.0), MI (0.9), and stroke (0.7).

## 4. Discussion

This meta-analysis and NMA of 63 RCTs investigated the effect of statins and omega-3 supplementation as well as their comparative efficacy of for the prevention of cardiovascular events. TG and HDL-C independent effects of total statins and omega-3 supplementation on the prevention of total CVD were observed. Furthermore, the effects of total statins and omega-3 supplementation on CHD, MI, and stroke were independent of the lipid-lowering effects of TC, TG, HDL-C, and LDL-C. Although the RCTs examined diverse populations, the findings were generally consistent for the significant risk reduction associated with statins in total CVD, CHD, MI, and stroke; conversely, a beneficial effect on stroke prevention was not observed for omega-3 supplementation. The findings were generally consistent in separate subgroup analyses of statins and omega-3 supplementation or in combined analyses of statins and omega-3 supplementation in terms of total CVD and CHD. Moreover, evidence was robust according to the Dersimonian–Laird methodology of the random-effects model, which accounts for between-study variability when at least two individual RCTs are included in pooled analysis.

The NMA of 264,516 adults showed that most statins and omega-3 supplementation were effective in reducing total CVD, CHD, and MI risks compared to the control group. Fluvastatin, atorvastatin, pitavastatin, and rosuvastatin were found to have the greatest effects on reducing total CVD, CHD, MI, and stroke, respectively.

Several guidelines have recommended statins for the primary and secondary prevention of CVD [89]. Statins can be classified as weak statins (simvastatin and pravastatin, compounds found in nature), strong (atorvastatin, pitavastatin, and rosuvastatin, synthetic molecules), and normal (fluvastatin and lovastatin) based on their ability to reduce cholesterol levels [90]. Yebyo et al. recently investigated the primary prevention effects of specific statins by NMA, though the date of the literature search was restricted to between 2013 and 2018, and the effect on total CVD and CHD was not investigated [31]. Statins were generally found to be associated with risk reductions in nonfatal MI (RR = 0.62, 95% CI = 0.53–0.72) and nonfatal stroke (RR = 0.83, 95% CI = 0.53–0.72) but not fatal MI or fatal stroke. In the current study, we found that statins significantly lowered the risks of combined fatal and nonfatal MI and stroke.

Several actions of omega-3 that overlap with the mechanisms of the pleiotropic effects of statins, including endothelial function improvement and antioxidant, anti-inflammatory, and antithrombotic effects, have been proposed [91,92]. Nonetheless, a previous meta-analysis also demonstrated no significant effects of omega-3 supplementation on total CVD (RR = 0.99, 95% CI = 0.89–1.09) and MI (RR = 0.81, 95% CI = 0.65–1.01) [93]. Rizos et al. also reported a null result for the effect of omega-3 supplementation on MI (RR = 0.89, 95% CI = 0.76–1.04) [94]. Aung et al. specified fatal and nonfatal CHD and different types of stroke, and the findings were still non-significant [28]. In contrast, Hu et al. recently updated findings from large-sample RCTs [11], including the A Study of Cardiovascular Events in Diabetes (ASCEND) trial [87], Vitamin D and Omega-3 Trial (VITAL) [88], and Reduction of Cardiovascular Events in Icosapent Ethyl-Intervention Trial (REDUCE-IT) that omega-3 supplementation significantly reduced the risk of total CVD (RR = 0.95, 95% CI = 0.92–0.98), MI (RR = 0.88, 95% CI = 0.83–0.94), and CHD (RR = 0.93, 95% CI = 0.89–0.96) [86]. Regardless a null finding for omega-3 supplementation in the prevention of stroke has consistently been reported [11,93,94].

This study had some intrinsic limitations. First, we did not specify the effect of statins and omega-3 supplementation on primary and secondary prevention of event outcomes, and primary prevention may be defined differentially in various studies. For example, Yebyo et al. categorized the population with CVD history was less than 10% of the total sample size as primary prevention [31], Naci et al. accepted a percentage up to 20% [22]. Additionally, the time frame in the identification of CVD history differed among RCTs. Therefore, we assumed that the effects of CVD and MI histories as well as other risk factors, such as hypertension, diabetes, and smoking, might not be significant. Second, arm-based data considering the sample size and number of events were included in the final analysis. Although the time to outcome for survival data is important in evaluating the effect of the intervention, the findings were not based on the contrast-based data of relative estimates because the hazard ratio was not available for all outcomes and in all RCTs. Third, there were only a few head-to-head RCTs of a statin versus a statin [80,81,82,83,84,85] and no RCT of a statin versus omega-3 supplementation. Last, due to the limitation of data availability, meta-regression for each type of statin as well as for omega-3 supplementation could not be performed separately.

Despite the abovementioned limitations, this study has important methodological strengths. To our knowledge, this is the first indirect comparison of statins versus omega-3 supplementation borrowing direct evidence of statins versus controls and omega-3 supplementation versus controls to provide quantitative evidence for the prevention of CVD events. The study was designed to minimize heterogeneity among controls from different RCTs. Although Abdelhamid et al. reported comprehensive evidence from 79 RCTs of omega-3 supplementation for the primary and secondary prevention of CVD [27], several RCTs were excluded from our analysis because of inappropriate control groups. The control group, which was defined as those with a lower intake of omega-3, might inappropriately develop an intermediary association with statins. RCTs in which the control group contained omega-6 were also excluded, as the debated effect of omega-6 intake has been reported [95,96,97,98,99]; thus, the inclusion of omega-6 in the control group might underestimate or overestimate the protective effect of omega-3. Moreover, robust findings were obtained by using different models in the meta-analysis and by comparing direct evidence from the meta-analysis and combined evidence from the NMA.

## 5. Conclusions

In summary, this study suggests that pravastatin and atorvastatin are more beneficial than omega-3 supplementation in reducing the risk of total CVD, CHD, and MI. Fluvastatin, atorvastatin, pitavastatin, and rosuvastatin showed the greatest effects on reducing total CVD, CHD, MI, and stroke, respectively.

## Figures and Tables

**Table 1:** Characteristics of trials included in the final analysis.

Study	Age (Years)	Follow-Up (Years)	MI (%)	Hypertension (%)	Diabetes (%)	Smoker (%)	Intervention Arm						Comparison Arm					
							Treatment	*N*	Total CVD	CHD	MI	Stroke	Treatment	*N*	Total CVD	CHD	MI	Stroke
Manson 2019 (VITAL) [88]	67.1	5.3		49.8	13.7	7.2	EPA+DHA	12,933	308	145	148	527	Control	12,938	370	200	142	567
Bhatt 2019 (REDUCE-IT) [86]	64	4.9			58.6		Icosapent Ethyl	4069		250			Control	4090		355		
Bowman 2018 (ASCEND) [87]	63.3	7.4	0	49.4	100	53.8	EPA+DHA	7740		186	252		Control	7740		200	251	
Yusuf 2016 (HOPE-3) [71]	63.8	5.6	0	37.9	5.8	27.7	Rosuvastatin	6361		105	45	70	Control	6344		140	69	99
Ford 2016 (WOSCOPS) [33]	55	4.9	0			44	Pravastatin	3302				194	Control	3293				223
Izawa 2015 (ALPS-AMI) [82]	66	2				63.2	Pravastatin	253				5	Atorvastatin	255				1
Hosomi 2015 (J-STAR) [32]		4.9		76	23.4	53.6	Pravastatin	780			5		Control	785			12	
Bonds 2014 (AREDS2) [79]	74	4.8	7	5.9	13	56.6	EPA+DHA	2147	183	28	28	48	Control	2056	187	30	30	41
Takano 2013 (PEARL) [43]	62.6	3	25.1	45.3	27.4		Pitavastatin	288			3	8	Control	286			8	9
Roncaglioni 2013 (Risk & Prevention) [52]	64	5		84.6	59.9	21.7	EPA+DHA	6239	620	310	80	80	Control	6266	630	324	90	60
Macchia 2013 (FORWARD) [14]	66.1			91.4	12.9	42.2	EPA+DHA	289	4		1	3	Control	297	4		1	3
Yakusevich 2012 [35]	65.7	1	9.8	77.6	9.8		Simvastatin	86			4	5	Control	97			5	7
Bosch 2012 (ORIGIN) [75]	63.5	6.2		79.5		12.4	EPA+DHA	6281	2055		344	314	Control	6255	2087		316	336
Nicholls 2011 (SATURN) [83]	57.6	2	24.4	70.4	15.3	32.3	Atorvastatin	519			11	2	Rosuvastatin	520			11	3
Emberson 2011 (MRC/BHF) [70]	64	5	43.5		30.4	74.8	Simvastatin	10,269				444	Control	10,267				585
Ostadal 2010 (FACS) [62]	62.1	1	7.7	51.3	19.2	29.2	Fluvastatin	78	10		2	1	Control	78	21		4	3
Kromhout 2010 (AlphaOmega) [60]	69	3.4	100	89.7	21	16.9	EPA+DHA	2404	170	122	89	11	Control	2433	185	132	102	10
Galan 2010 (SU.FOL.OM3) [67]	61.4	4.7	46			72.8	EPA+DHA	1253	81	51	32	29	Control	1248	76	53	28	28
Einvik 2010 (DO IT) [66]	70	3		28	14.5	34	EPA+DHA	282	32	11	11		Control	281	36	9	9	
Dangour 2010 (OPAL) [13]	75	2	3.9	55.9			EPA+DHA	434				7	Control	433				8
Chan 2010 (ASTRONOMER) [34]	58	3.5				48.3	Rosuvastatin	134	35				Control	135	44			
Rauch 2009 (OMEGA) [53]	64	1	14.4	66.5	27	36.7	EPA+DHA	1919	199	547	87	27	Control	1885	165	568	78	13
Mok 2009 (RCASS) [65]	63	2		69.2	91.2	26.4	Simvastatin	113		2			Control	114		3		
Fellstrom 2009 (AURORA) [33]	64.2	3.8	10.2		26.4	15.5	Rosuvastatin	1389			91	53	Control	1384			107	45
Tavazzi 2008 (GISSI-HF) [45]	68	3.9	14.2	54.6	28.3	14.2	EPA+DHA	3494	1635		107	122	Control	3481	1687		129	103
Tavazzi 2008 (GISSI-HF) [37]	68	3.9		54.3		14.1	Atorvastatin	2285			61	82	Control	2289			70	66
Ridker 2008 (JUPITER) [47]	66	1.9	0			15.8	Rosuvastatin	8901			31	33	Control	8901			69	64
Yokoyama 2007 (JELIS) [36]	61	4.6	5.6	35.5	16.3	18.9	EPA	9326		262	71	166	Control	9319		324	93	162
Kjekshus 2007 (CORONA) [55]	73	2.7	60	63	29.5	8.6	Rosuvastatin	2514			131	126	Control	2497			154	138
Deedwania 2007 (SAGE) [81]	72.5	1	45.9	64.5	23.2	59.4	Atorvastatin	446			16	1	Pravastatin	445			16	3
Nakamura 2006 (MEGA) [63]	58.3	5.3		41.8	20.8	20.6	Pravastatin	3866	125	66	17	50	Control	3966	172	101	33	62
Knopp 2006 (ASPEN) [59]	61	4	16.4	55.1	100	12.4	Atorvastatin	1211			297		Control	1199			313	
Brouwer 2006 (SOFA) [12]	61.5	1	62.6	50.7	15.9	67	EPA+DHA	273	65	1			Control	273	62	3		
Amarenco 2006 (SPARCL) [77]	62.7	4.9				59	Atorvastatin	2365	530	123	43	265	Control	2366	687	204	82	311
Yokoi 2005 (ATHEROMA) [40]	59.3	3	45.5	42	18.8		Pravastatin	142	23		2	5	Control	146	25		4	4
Wanner 2005 [41]	65.7	4	17.6		100	40.4	Atorvastatin	619	205		93	60	Control	636	246		112	45
Stone 2005 [42]		1	38.9	62.4	16.5	69.5	Atorvastatin	96			1		Control	103			1	
Raitt 2005 [16]	62.5	2	55.5	50.5			EPA+DHA	100	2	1	1		Control	100	5	3	3	
Pedersen 2005 (IDEAL) [85]	61.7	4.8	28	58.4	12	79.1	Atorvastatin	4439	1176	898	267	151	Simvastatin	4449	1370	1059	321	174
Makuuchi 2005 (PCABG) [54]	58.9	4.5	62	51.5	33.3	41.9	Pravastatin	152	26		1	4	Control	151	36		4	3
Sever 2004 (ASCOT-LLA) [46]	63	5		100	24.6	32.7	Atorvastatin	5168	389			89	Control	5137	486			121
Nissen 2004 (REVERSAL) [84]	56.2	1.5		68.5	18.9	26.3	Atorvastatin	253			4	1	Pravastatin	249			7	1
Nakagawa 2004 (PCS) [64]	55	5.4		59.2	17.5	67.5	Pravastatin	54	13		2	3	Control	66	19		3	4
Koren 2004 (ALLIANCE) [58]	61.2	4.5	57.8		22.1	19.5	Atorvastatin	1217	408		52	35	Control	1225	443		94	39
Colhoun 2004 (CARDS) [74]	62	3.9			100	65.3	Atorvastatin	1428				21	Control	1410				39
Cannon 2004 (PROVE IT-TIMI) [80]	58.2	2	18.5	50.2	17.6		Atorvastatin	2099			139	21	Pravastatin	2063			153	21
Shepherd 2002 (PROSPER) [49]	75.3	3.2	13.4	61.9	10.7	26.8	Pravastatin	2891	454	77		135	Control	2913	523	102		131
Serruys 2002 (LIPS) [50]	60	3.9	44.4	38.6	12	71.5	Fluvastatin	844				2	Control	833				1
Sawayama 2002 (FAST) [51]	66.3	2		39.6	25	57.8	Pravastatin	83	4		4		Control	81	11		11	
Liem 2002 (FLORIDA) [56]	60.5	1	11.5				Fluvastatin	265			21		Control	275			13	
Davis 2002 (ALLHAT-LTT) [73]	66.4	4.8			35.1	23.2	Pravastatin	5170				209	Control	5185				231
Arthros 2002 (GREACE) [78]	58.5	3	81.2	42.9	19.6		Atorvastatin	800			21	9	Control	800			51	17
Nilsen 2001 [15]	64	1.5	23.3	24.3	10.3	75.7	EPA+DHA	150	42		17		Control	150	36		12	
Teo 2000 (SCAT) [39]	61	4	70	36	11	82	Simvastatin	230			11	4	Control	230			10	7
Valagussa 1999 (GISSI-P) [44]	59.4	3.5	12	35.6	14.8	77.2	EPA+DHA	2836				44	Control	2828				41
Riegger 1999 [48]	59.8	1	35.6	29.3	5.5	9.6	Fluvastatin	187					Control	178				
Plehn 1999 (CARE) [57]		5	100	42.7	14.1	16.1	Pravastatin	2081				92	Control	2078				124
Tonkin 1998 (LIPID) [38]	62	6.1		41.7	8.7	73.3	Pravastatin	4512			336	169	Control	4502			463	204
Downs 1998 (AFCAPS/TexCAPS) [72]	58	5.2		21.9	2.3	12.4	Lovastatin	3304	194	163	57		Control	3301	255	215	95	
Bestehorn 1997 (CIS) [76]	49.8	2.3				84.3	Simvastatin	129			1		Control	125			5	
Jukema 1995 (REGRESS) [61]	56	2	47.4	27.8	0.1	88	Pravastatin	450	59				Control	434	93			
Furberg 1995 (PLAC-I & II) [68]	58	3	48.5	40.5	0.7	15.5	Pravastatin	281		14	9		Control	278		29	24	
Furberg 1994 (ACAPS) [69]	62	2.8		28.8		56.5	Lovastatin	460			5		Control	459			5	

CVD, cardiovascular disease; CHD, coronary heart disease; MI, myocardial infarction; EPA, eicosapentaenoic acid; and DHA, docosahexaenoic acid.

**Table 2:** Lipid profiles of study participants.

Study	Baseline Measurement								Change Difference			
	Intervention Arm				Comparison Arm							
	TC (mg/dL)	TG (mg/dL)	HDL-C (mg/dL)	LDL-C (mg/dL)	TC (mg/dL)	TG (mg/dL)	HDL-C (mg/dL)	LDL-C (mg/dL)	TC (mg/dL)	TG (mg/dL)	HDL-C (mg/dL)	LDL-C (mg/dL)
Manson 2019 (VITAL) [88]												
Bhatt 2019 (REDUCE-IT) [86]		216.5	40.0	74.0		216.0	40.0	76.0		−32.5	0	−5.0
Bowman 2018 (ASCEND) [87]												
Yusuf 2016 (HOPE-3) [71]	201.5	128.8	44.7	127.8	201.3	126.5	44.9	127.9		−21.2		−34.6
Ford 2016 (WOSCOPS) [33]	272.0	162.0	44.0	192.0	272.0	164.0	44.0	192.0				
Izawa 2015 (ALPS-AMI) [82]	204.1	142.9	47.6	130.2	203.2	130.8	48.0	131.0			−0.6	
Hosomi 2015 (J-STAR) [32]	210.8	142.6	53.8	129.5	209.6	141.7	53.0	129.5	−23.2	−9.7	0.8	−21.3
Bonds 2014 (AREDS2) [79]												
Takano 2013 (PEARL) [43]	203.2		50.7	125.2	201.2		50.8	125.5			2.0	−31.5
Roncaglioni 2013 (Risk & Prevention) [52]	215.6	150.0	50.9	131.8	216.4	150.0	51.2	132.5	−0.5	−8.1	0.6	−0.4
Macchia 2013 (FORWARD) [14]												
Yakusevich 2012 [35]	211.5	102.7		85.1	201.9	91.2		86.2	−29.4	−11.5		8.9
Bosch 2012 (ORIGIN) [75]	189.0		46.0	112.0	190.0		46.0	112.0	−1.1	−14.5	0.1	0.6
Nicholls 2011 (SATURN) [83]	193.5	130.0	44.7	119.9	144.1	110.0	48.6	70.2	−44.7	−30.0	2.1	−42.1
Emberson 2011 (MRC/BHF) [70]												−32.9
Ostadal 2010 (FACS) [62]	212.7				208.8				−56.2			
Kromhout 2010 (AlphaOmega) [60]	182.5	145.3	49.9	110.2	182.9	147.9	49.5	110.2				
Galan 2010 (SU.FOL.OM3) [67]	174.0	106.3	46.4	104.4	175.9	106.3	46.4	102.5				
Einvik 2010 (DO IT) [66]	243.6	150.6	54.1	158.1	239.8	150.6	54.1	154.7				
Dangour 2010 (OPAL) [13]												
Chan 2010 (ASTRONOMER) [34]	206.1	108.9	61.5	123.0	203.8	116.9	59.9	120.6			1.9	−66.9
Rauch 2009 (OMEGA) [53]												
Mok 2009 (RCASS) [65]	226.2	118.7	45.6	151.6	227.0	125.8	44.9	150.4	−54.5	−11.5		−50.7
Fellstrom 2009 (AURORA) [33]	176.0	157.0	45.0	100.0	174.0	154.0	45.0	99.0	−46	−24.2	0	−40.1
Tavazzi 2008 (GISSI-HF) [45]												
Tavazzi 2008 (GISSI-HF) [37]				122.2				121.0				−0.1
Ridker 2008 (JUPITER) [47]	186.0	118.0	49.0	108.0	185.0	118.0	49.0	108.0		−19.0	0	−54.0
Yokoyama 2007 (JELIS) [36]	274.9	153.2	58.8	181.4	274.9	154.1	58.4	181.7	0	−7.6		0
Kjekshus 2007 (CORONA) [55]		178.0	48.0	137.0		176.0	47.0	136.0		−42.0	2.0	−63.0
Deedwania 2007 (SAGE) [81]	225.8	164.4	45.5	147.5	221.9	157.1	46.4	144.0	−18.3	−19.3	−2.6	−23.0
Nakamura 2006 (MEGA) [63]	242.5	127.5	57.6	156.6	242.5	127.5	57.6	156.6	−20.5	−14.2	19.3	−20.7
Knopp 2006 (ASPEN) [59]	194.0	147.0	47.0	113.0	194.0	145.0	47.0	114.0	−35.5	−20.2	1.1	−33.0
Brouwer 2006 (SOFA) [12]												
Amarenco 2006 (SPARCL) [77]	211.4	144.2	50.0	132.7	212.3	143.2	50.0	133.7	−60.3	−34.5	1.1	−54.6
Yokoi 2005 (ATHEROMA) [40]	226.2	181.1	49.1	143.3	224.8	167.1	50.0	142.0	−27.8	−14.2	0.5	−26.7
Wanner 2005 [41]	218.0	261.0	36.0	127.0	220.0	267.0	36.0	125.0				−50.0
Stone 2005 [42]	228.0	149.0	44.0	149.0	230.0	151.0	43.0	151.0				−35.9
Raitt 2005 [16]												
Pedersen 2005 (IDEAL) [85]	196.8	151.1	46.0	121.6	195.9	146.6	46.1	121.4	−24.3	−23.2	−0.4	−20.0
Makuuchi 2005 (PCABG) [54]	213.7	166.3	41.4	141.4	214.4	154.2	41.3	141.1	−29.3	−30.5	2.4	−20.3
Sever 2004 (ASCOT-LLA) [46]	211.9	147.0	50.7	133.0	211.9	146.1	50.7	133.0	−38.7	−18.6	0.8	0
Nissen 2004 (REVERSAL) [84]	231.8	197.2	42.3	150.2	232.6	197.7	42.9	150.2	−35.4	−16.9	−0.9	−31.5
Nakagawa 2004 (PCS) [64]	200.1	141.9	43.0	128.7	200.5	143.9	43.3	128.3	−17	−7.9	0.4	−15.3
Koren 2004 (ALLIANCE) [58]	226.0	197.0	40.0	147.0	225.0	198.0	41.0	146.0	−20.0	−12.0	0	−16.0
Colhoun 2004 (CARDS) [74]	207.3	150.6	53.8	117.6	206.9	147.9	54.9	116.8	−45.3	−28.4	2.3	−39.9
Cannon 2004 (PROVE IT-TIMI) [80]	181.0	158.0	38.0	106.0	180.0	154.0	39.0	106.0			0.3	−33.0
Shepherd 2002 (PROSPER) [49]	220.4	132.9	50.3	146.9	220.4	132.9	50.3	146.9				−34.8
Serruys 2002 (LIPS) [50]	200.0	160.0	38.0	131.0	199.0	160.0	37.0	132.0	−1.0	0	0.3	−49.9
Sawayama 2002 (FAST) [51]	251.5	168.7	56.7	160.7	255.2	135.8	56.5	171.6	−1.8		15.3	−43.1
Liem 2002 (FLORIDA) [56]	204.9	150.6	46.4	135.3	208.8	141.7	46.4	139.2	−45.4	−29.2	1.8	−40.9
Davis 2002 (ALLHAT-LTT) [73]	223.7	150.6	47.6	145.6	223.7	152.8	47.4	145.5	−18.9	0.2	3.4	−17.3
Arthros 2002 (GREACE) [78]	257.0	184.0	39.0	180.0	255.0	178.0	39.0	179.0	−82.0	−56.0	2.0	−73.0
Nilsen 2001 [15]	229.7	145.3	41.8		231.6	137.3	44.9		−11.3	−52.9	4.8	
Teo 2000 (SCAT) [39]	202.2	163.9	38.3	131.1	198.4	156.8	37.5	128.8	−48.0	−31.9	1.9	−44.1
Valagussa 1999 (GISSI-P) [44]	210.2	162.6	41.5	137.3	211.6	161.9	41.7	138.5	2.6	−6.3	−0.2	4.2
Riegger 1999 [48]	289.0	189.0	53.0	198.0	284.0	183.0	56.0	193.0	−36.4			−37.9
Plehn 1999 (CARE) [57]	209.0	156.0	39.0	139.0	209.0	155.0	39.0	139.0	42.0	22.0	−2.0	44.0
Tonkin 1998 (LIPID) [38]	218.0	142.0	36.0	150.0	218.0	138.0	36.0	150.0				
Downs 1998 (AFCAPS/TexCAPS) [72]	225.4	168.2	36.8	153.3	209.2	166.8	37.5	153.6	−60.3	−29.0	1.7	−40.6
Bestehorn 1997 (CIS) [76]	240.3		44.3	164.5	243.4		43.6	167.4				
Jukema 1995 (REGRESS) [61]	232.8	156.8	36.0	166.3	234.0	159.4	36.0	166.7	−45.2	−19.5	3.1	−44.5
Furberg 1995 (PLAC-I & II) [68]	232.0	166.0	41.0	165.0	230.0	169.0	41.0	162.0				
Furberg 1994 (ACAPS) [69]	235.2		51.7	156.5	235.3		52.2	154.6				

CVD, cardiovascular disease; CHD, coronary heart disease; MI, myocardial infarction; EPA, eicosapentaenoic acid; DHA, docosahexaenoic acid; TC, total cholesterol; TG, triglyceride; HDL-C, high-density lipoprotein cholesterol; LDL-C, low-density lipoprotein cholesterol. Data are presented as the mean or median serum lipid concentration. Change difference is defined as difference between lipid changes in the intervention arm versus lipid changes in the comparison arm. Change difference = (achieved level—baseline level) _intervention arm_—(achieved level—baseline level) _comparison arm_.

**Table 3:** Pooled relative risk and 95% confidence interval from meta-analyses.

	*N* (I^2^)	Fixed-Effects Model	Random-Effects Model
**Total cardiovascular** **disease**			
Statins	14 (30.0%)	0.81 (0.78–0.85)	0.81 (0.76–0.86)
Atorvastatin	4 (55.5%)	0.83 (0.78–0.88)	0.83 (0.76–0.91)
Fluvastatin	1 (NA)	0.48 (0.24–0.94)	0.48 (0.24–0.94)
Lovastatin	1 (NA)	0.76 (0.63–0.91)	0.76 (0.63–0.91)
Pravastatin	7 (28.8%)	0.81 (0.74–0.89)	0.77 (0.67–0.89)
Rosuvastatin	1 (NA)	0.80 (0.55–1.16)	0.80 (0.55–1.16)
Omega-3	13 (0%)	0.98 (0.95–1.01)	0.98 (0.95–1.01)
Overall	27 (59.6%)	0.92 (0.90–0.94)	0.89 (0.85–0.94)
**Coronary heart disease**			
Statins	7 (0%)	0.69 (0.62–0.77)	0.70 (0.62–0.77)
Atorvastatin	1 (NA)	0.60 (0.49–0.75)	0.60 (0.49–0.75)
Lovastatin	1 (NA)	0.76 (0.62–0.92)	0.76 (0.62–0.92)
Pravastatin	3 (0%)	0.69 (0.56–0.84)	0.69 (0.56–0.84)
Rosuvastatin	1 (NA)	0.75 (0.58–0.96)	0.75 (0.58–0.96)
Simvastatin	1 (NA)	0.67 (0.11–3.95)	0.67 (0.11–3.95)
Omega-3	10 (0%)	0.90 (0.84–0.96)	0.90 (0.85–0.96)
Overall	17 (44.6%)	0.84 (0.79–0.88)	0.81 (0.75–0.89)
**Myoca** **rdial infarction**			
Statins	27 (51.4%)	0.74 (0.69–0.79)	0.69 (0.61–0.78)
Atorvastatin	7 (73.6%)	0.79 (0.71–0.87)	0.70 (0.55–0.89)
Fluvastatin	2 (42.6%)	1.40 (0.76–2.56)	1.18 (0.40–3.46)
Lovastatin	2 (0%)	0.62 (0.45–0.85)	0.62 (0.45–0.85)
Pitavastatin	1 (NA)	0.37 (0.10–1.39)	0.37 (0.10–1.39)
Pravastatin	8 (3.1%)	0.68 (0.60–0.77)	0.66 (0.56–0.78)
Rosuvastatin	4 (62.9%)	0.74 (0.64–0.86)	0.71 (0.55–0.91)
Simvastatin	3 (11.2%)	0.82 (0.43–1.56)	0.85 (0.41–1.78)
Omega-3	15 (45.6%)	0.88 (0.82–0.94)	0.89 (0.80–0.99)
Overall	42 (53.8%)	0.81 (0.77–0.85)	0.78 (0.72–0.85)
**Stroke**			
Statins	26 (33.3%)	0.84 (0.80–0.89)	0.85 (0.79–0.92)
Atorvastatin	7 (64.9%)	0.88 (0.79–0.98)	0.88 (0.71–1.10)
Fluvastatin	2 (11.4%)	0.75 (0.17–3.31)	0.77 (0.13–4.38)
Pravastatin	9 (0%)	0.88 (0.80–0.96)	0.88 (0.80–0.96)
Rosuvastatin	4 (68.8%)	0.81 (0.70–0.95)	0.80 (0.60–1.07)
Simvastatin	3 (0%)	0.76 (0.67–0.85)	0.76 (0.67–0.85)
Omega-3	13 (36.1%)	1.02 (0.94–1.10)	0.88 (0.71–1.10)
Overall	39 (46.2%)	0.90 (0.86–0.94)	0.91 (0.85–0.98)

**Table 4:** Change difference in lipid profiles in the association with risk reduction of cardiovascular events.

Covariate	Total Cardiovascular Disease		Coronary Heart Disease		Myocardial Infarction		Stroke	
	Coefficient	*p*-Value	Coefficient	*p*-Value	Coefficient	*p*-Value	Coefficient	*p*-Value
TC	0.0046	<0.001	0.0042	0.047	0.0055	0.08	−0.0111	0.53
TG	0.0026	0.51	0.0098	0.004	0.0009	0.89	−0.0117	0.67
HDL-C	−0.0116	0.18	−0.0065	0.64	−0.0198	0.35	0.0480	0.67
LDL-C	0.0034	0.004	0.0044	0.048	0.0048	0.09	−0.0126	0.36

TC, total cholesterol; TG, triglyceride; HDL-C, high-density lipoprotein cholesterol; LDL-C, low-density lipoprotein cholesterol.

**Table 5:** Ranking probabilities and surface under the cumulative ranking (SUCRA) values of treatments according to different outcomes.

	1st	2nd	3rd	4th	5th	6th	7th	8th	9th	SUCRA
**Total cardiovascular** **disease**										
Control	0	0	0	0	0.3	5.3	33.2	61.3		0.1
Omega-3	0	0	0	0.2	7.5	43.3	44.3	4.7		0.2
Simvastatin	0	0	0.1	0.8	13.2	45.5	19.6	20.7		0.2
Pravastatin	0.7	11.4	27.2	34.6	25.8	0.3	0	0		0.6
Fluvastatin	85.4	7.1	1.7	1.5	2.1	0.6	0.3	1.3		0.9
Lovastatin	3.6	32.0	30.1	17.5	15.6	1.0	0.2	0.1		0.7
Atorvastatin	0.7	11.3	31.5	37.5	19.0	0	0	0		0.6
Rosuvastatin	9.7	38.3	9.5	7.9	16.5	3.9	2.4	11.9		0.6
**Coronary heart disease**										
Control	0	0	0	0	0	2.5	97.5			0
Omega-3	0	0	0.1	1.2	14.8	83.3	0.7			0.2
Simvastatin	0	26.8	26.1	24.6	18.8	3.4	0.3			0.6
Pravastatin	13.3	31.6	28.0	17.9	8.8	0.4	0			0.7
Lovastatin	3.2	12.3	21.7	29.5	29.1	3.9	0.3			0.5
Atorvastatin	78.1	15.9	4.9	1.2	0	0	0			1
Rosuvastatin	5.4	13.5	19.4	25.6	28.6	6.4	1.2			0.5
**Myocardial infarction**										
Control	0	0	0	0	0.1	1.4	18.8	66.5	13.2	0.1
Omega-3	0	0	0.1	0.8	7.4	33.0	49.5	8.5	0.7	0.3
Simvastatin	1.0	3.7	5.8	10.1	18.0	29.2	17.4	11.6	3.3	0.4
Pravastatin	8.4	30.1	28.2	19.0	10.6	3.3	0.4	0	0	0.7
Fluvastatin	0.2	0.8	0.7	0.9	2.0	3.6	5.4	7.4	78.9	0.1
Lovastatin	13.3	32.8	13.5	12.0	13.4	9.3	3.7	1.8	0.3	0.7
Atorvastatin	2.8	16.0	30.9	30.9	16.5	2.7	0.2	0	0	0.7
Pitavastatin	72.7	6.0	2.9	2.7	3.5	3.0	2.1	4.0	3.6	0.9
Rosuvastatin	2.1	10.6	17.9	23.6	28.5	14.4	2.6	0.3	0	0.6
**Stroke**										
Control	0	0	0.1	1.2	13.3	38.1	37.2	10.1		0.2
Omega-3	0	0	0.4	2.3	8.0	21.7	39.6	28.0		0.2
Simvastatin	10.9	23.4	23.1	19.5	13.0	6.6	2.4	1.1		0.7
Pravastatin	1.9	8.0	16.8	27.3	28.2	14.7	2.2	0.7		0.5
Fluvastatin	42.2	8.2	2.9	2.6	3.4	3.0	6.1	31.5		0.6
Atorvastatin	5.5	18.2	28.3	25.2	16.3	5.6	0.6	0.2		0.6
Pitavastatin	25.9	15.5	4.6	4.7	5.5	5.2	10.6	28.0		0.5
Rosuvastatin	13.5	26.6	23.7	17.1	12.3	5.2	1.2	0.4		0.7
